# Paradoxical Vocal Cord Motion Presaging Bilateral Vocal Cord Paresis in an Infant

**DOI:** 10.7759/cureus.7853

**Published:** 2020-04-27

**Authors:** Chelsea N Cleveland, Allyson Miller, Cesar A Serrano, Michele M Carr

**Affiliations:** 1 Otolaryngology, Jacobs School of Medicine and Biomedical Sciences, University at Buffalo, Buffalo, USA; 2 Otolaryngology, Larner College of Medicine at the University of Vermont, Burlington, USA; 3 Neurosurgery, West Virginia University, Morgantown, USA

**Keywords:** chiari i malformation, paradoxical vocal cord motion, bilateral vocal cord paralysis, hydrocephalus, vp shunt, arnold chiari malformation, infantile, chiari ii malformation

## Abstract

Paradoxical vocal cord motion (PVCM) is a condition characterized by inappropriate adduction of the vocal cords during respiration. Usually seen in children and adolescents, PVCM presentation in infants is uncommon. Once thought to be a product of psychiatric disease, there are now several other proposed etiologies including irritant-induced and secondary to neurologic disease. Previous studies showed that the treatment of gastric reflux in this age group leads to a resolution of symptoms. We present a case of PVCM in an infant with hydrocephalus and Chiari II malformation. She received reflux therapy and ventriculoperitoneal (VP) shunting with two revisions. Despite these interventions, she continued with symptoms and eventually progressed to bilateral vocal cord paralysis (VCP). There is a paucity of literature describing PVCM as a precursor to VCP. Clinicians should be aware that in this population, refractory PVCM may serve as a warning sign for further vocal cord function decline.

## Introduction

Paradoxical vocal cord motion (PVCM) is episodic inappropriate adduction of the vocal cords during respiration [1-2]. Predominantly a disease of children and adolescents, there are few reports of infants with PVCM [3-4]. Formerly thought to be solely a psychiatric manifestation, this phenomenon has been described in those with neurological diseases and as a result of vocal cord irritation. Reflux therapy in infants has shown resolution of symptoms of PVCM [5-10]. Bilateral vocal cord paralysis (VCP) in which there is a cessation of movement in both vocal folds can also be caused by neurologic diseases in children [11-15]. There have been no reports of refractory PVCM progressing to VCP. Here, we present a case of an infant with a Chiari II malformation who presented with PVCM with subsequent VCP.

## Case presentation

A term female born to a 26-year-old mother by cesarean section had a birth weight of 3.45 kg (>75th percentile) with a head circumference of 44 cm (>98th percentile). Her one-minute Apgar was 3 with peripheral cyanosis, heart rate of 70 to 100 beats per minute, aphonia, and poor tone. Positive pressure ventilation was started after which her heart rate normalized. Crying and grimacing ensued. She did not require chest compressions or epinephrine. She continued to have an abnormal breathing pattern and decreased tone along with persistent peripheral cyanosis at five minutes with an Apgar of 8. She was taken to the neonatal intensive care unit (NICU) where the abnormal breathing pattern and grunting persisted. She was intubated and ventilated.

**Figure 1:**
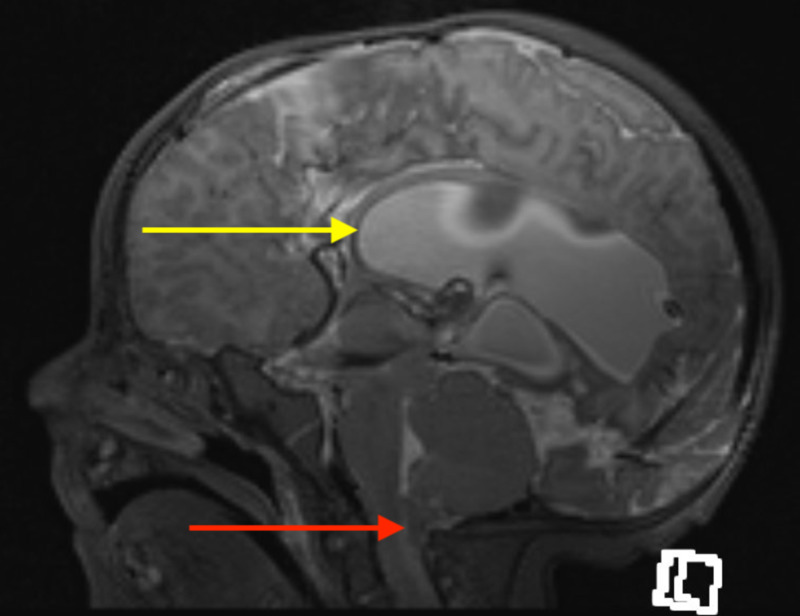
T2 sagittal MRI showing a Chiari II malformation (red arrow) and enlarged ventricles (yellow arrow) MRI: magnetic resonance imaging

She had significant dolichocephaly, congenital hypothyroidism, bilateral club foot, hydrocephalus, and myelomeningocele. She had flaccidity and no response to touch in the lower limbs. Magnetic resonance imaging (MRI) showed dolichocephaly with marked hydrocephalus and colpocephaly (Figure 1). The corpus callosum was not visualized. Cerebral encephalomalacia, with ectopia of the cerebellum, and a Chiari II malformation were present. She underwent right ventriculoperitoneal (VP) shunt and myelomeningocele repair at Day 1 of life. She was discharged from the hospital on a pulse oximeter at 15 days of life at which point she was stable on room air and taking oral feeds with limited weight gain. She had a strong cry and there was no noisy breathing or increased respiratory effort noted.

At one month of age, she presented to the emergency department (ED) with difficulty breathing. Mom reported that the baby had “sounded stuffy since discharge." This was initially associated with feeds and had progressed to being continually present. There was associated sternal indrawing, subcostal retractions, back arching, and suctioning of feeds and mucus from her nose. There was minimal vomiting, occurring mainly after feeds. Since the otolaryngology team perceived significant stridor, she underwent flexible laryngoscopy. This showed a posteriorly positioned tongue base, retroflexed epiglottis, short aryepiglottic folds, and post-glottic edema that collapsed into the airway, consistent with laryngomalacia. The vocal cords were not visualized due to laryngeal tissue collapse. A chest X-ray and modified barium swallow were normal. A repeat MRI showed no changes. Polysomnogram showed a central apnea index of 21.5, obstructive apnea index of 8, oxygen saturation nadir of 82% (which was <90% for <1% of recording), and normal transcutaneous CO_2_. Since flexible laryngoscopy showed a significant obstruction of the larynx while awake, she underwent direct laryngoscopy and bronchoscopy and had a supraglottoplasty performed on the first day of admission. She was on lansoprazole as an inpatient but was discharged on ranitidine at 3 mg/kg/day.

She did well with quiet breathing and good feeding for about a month before the stridor recurred. She had increasing difficulty feeding. At this point, flexible laryngoscopy showed paradoxical vocal cord movement (PVCM) in the supine and vertical positions. The vocal cord adduction was coincident with audible stridor. Her vocal cords clearly opened intermittently. The epiglottis was still retro-positioned by the tongue base and there was post-glottic and vocal cord edema with posterior pharyngeal wall cobblestoning, but no resulting airway obstruction. At this point, lansoprazole 7.5 mg/day was added to ranitidine 3 mg/kg/day. She was assessed by pulmonology but management was not changed.

At four months old, MRI suggested shunt failure with significant enlargement of the cortical mantle, small ventricles, Chiari II persistence, and new syrinx. The shunt failure, abnormal vocal cord movement, and decline in feeding instigated a shunt revision. She did well during and after surgery and was extubated the following day. She was discharged on ranitidine 6 mg/kg/day. One week after surgery, flexible laryngoscopy again demonstrated episodic PVCM. Several normal breaths with normal vocal cord opening were seen. One week later, a second sleep study showed an obstructive Apnea-hypopnea index (AHI) of 20.56, a central index of 4.55, and an oxygen saturation nadir of 73%. Transcutaneous CO_2_ values were not significantly elevated. Repeat swallow study did not demonstrate aspiration but did show esophageal retention and retrograde flow in the mid-esophagus. Her parents refused tracheotomy at this point, which was recommended for her severe obstructive sleep apnea (OSA).

At six months of age, her mother reported improved sleep and feeding. Flexible laryngoscopy showed a few breaths with PVCM but mainly normal laryngeal respiration. There was evidence of mild laryngomalacia with the intermittent collapse of the posterior larynx into the airway. She was maintained on ranitidine 6 mg/kg/day. By eight months old, she was feeding better, gaining weight well, and developing along a delayed course. She underwent craniofacial evaluation and was prescribed a molding helmet, as all cranial sutures were still open. She underwent repeat flexible laryngoscopy with similar findings although her ranitidine had been stopped by her pediatrician.

At nine months of age, she was admitted for an episode of prolonged back arching and cyanosis that occurred during feeding, lasted less than a minute, and was followed by sleepiness. Per her mother, she was back to baseline after 10 minutes. An X-ray shunt series and an MRI did not suggest shunt malfunction. There was no papilledema. The electroencephalogram (EEG) was normal. A diagnosis of reflux episode with possible aspiration was made and she was discharged home with ranitidine 4 mg/kg/day.

At 11 months, she was admitted again with suspicion of febrile seizure. She had respiratory symptoms about three weeks prior. On the night of admission, she had a low-grade fever at home, shaking of her right arm and leg, rhythmic mouth movements, sialorrhea, and she was unresponsive, with her eyes open. She was given 1 mg intramuscular midazolam and had a very short seizure-like episode after that, which resolved rapidly. She was given 200 mg of fosphenytoin and acetaminophen. She was transferred to the pediatric intensive care unit (PICU) where she had a mildly elevated white blood cell count at 15.6/uL. Again, a shunt series X-ray showed no obvious shunt malfunction. EEG showed left focal slowing. She was discharged on oxcarbazepine 60 mg bid.

**Figure 2:**
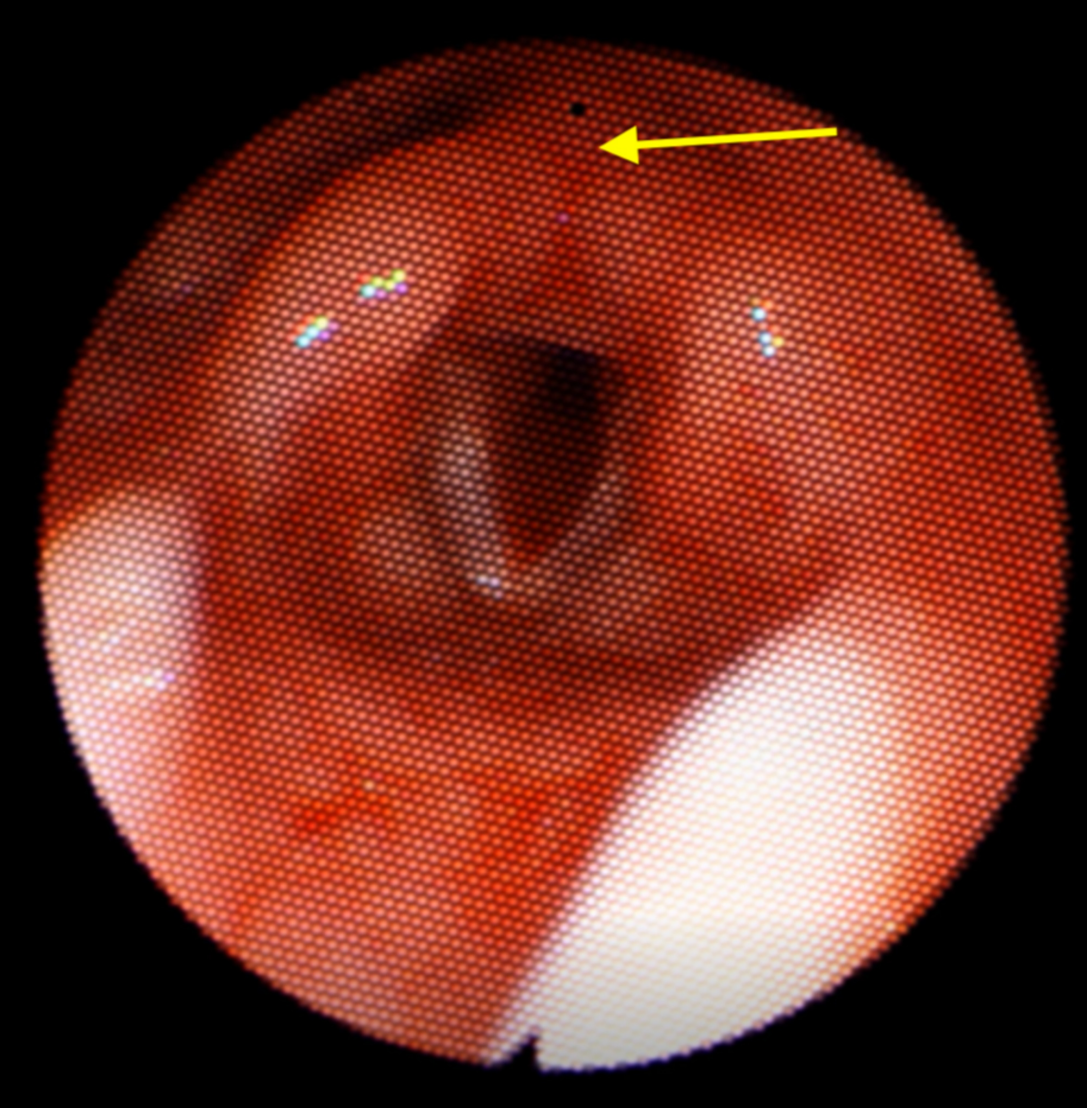
Flexible laryngoscopy after supraglottoplasty revealing mild post-glottic edema, well-healed aryepiglottic folds, and well-positioned epiglottis with patent glottic aperture.

Just before her first birthday, she continued to have OSA with desaturations at night despite oxygen supplementation so she underwent sleep endoscopy (Figure 2). This showed multilevel narrowing of the airway but no complete obstruction. She had a circumferential collapse of her airway at the palate/tonsil level, retro-lingual narrowing, and PVCM was demonstrated on a few breaths. Pulmonology recommended continued oxygen supplementation as continuous positive airway pressure (CPAP) mask fitting was impossible because of the shape of her head.

At 15 months old, she was readmitted for episodes that her parents described as “spacing out” with perioral cyanosis, mouth clenching, followed by flaccid spells. They stated it looked like she was "choking on something" but there was nothing in her mouth. They estimated one episode a week for the past month. She had her oxcarbazepine dose increased, without improvement in the episodes. Two weeks later, she was seen in the otolaryngology clinic for follow-up. She was feeding well but had increased stridor and continued to have these episodes, which were considered to be possible reflux symptoms. Flexible laryngoscopy showed bilateral vocal cord immobility with a paramedian position, an open posterior chink, and post-glottic edema. Two months later, she underwent shunt revision as brain magnetic resonance imaging (MRI) showed enlargement of her ventricles. The shunt was found to be kinked proximally intraoperatively. A bronchoscopy performed one month after shunt revision revealed no evidence of bilateral vocal cord paresis. On follow up with pulmonology, she had no stridor. She had a near-normal sleep study with an AHI of 1.4 and one episode of oxygen desaturation. Her vocal cord symptoms improved following her shunt revision, and tracheostomy was avoided.

## Discussion

This patient showed paradoxical vocal cord motion (PVCM) associated with stridor, dysphagia, obstructive sleep apnea, a VP shunt, and a Chiari II malformation. Her cranial nerve symptoms worsened with shunt malfunction and improved but did not completely resolve with shunt revision. She has continued to have a Chiari malformation throughout her reported course. PVCM is not frequently reported in infants and has not been reported as a precursor to bilateral vocal cord paresis.

PVCM is considered to be inappropriate adduction of the vocal folds during respiration [1-2]. Traditionally, PVCM affects children and young adults with an average age at diagnosis of 14.5 years in the pediatric population [3]. There have been few reports of PVMC in infants [4]. Historically, vocal cord dysfunction has been attributed to be a psychological disorder rather than a physical abnormality. Several psychiatric conditions were implicated in the development of PVCM and the literature had largely concluded that it was a conversion reaction to emotional stress [5]. However, there are now several other proposed etiologies. 

Irritant associated PVCM occurs secondary to a reflexive closure of the vocal folds after exposure to an irritant such as in refluxed stomach contents. A literature review determined that 18% of patients with PVCM also have a diagnosis of reflux [6]. In children, there has been a resolution of PVCM symptoms with proton pump inhibitor use [4-5]. There have also been cases where PVCM occurs during sleep in children with an absence of daytime symptoms [7-8]. Many neurologic conditions have been associated with the development of PVCM including multisystem atrophy, brainstem compression, cortical injuries, and neuromuscular disorders [8-10].

The causative factors of bilateral vocal cord paralysis (VCP) in infants and children include neurologic, traumatic, iatrogenic, or idiopathic etiologies. The most common neurologic finding is Chiari malformation, diagnosed by MRI imaging, demonstrating cerebellar ectopia below the foramen magnum. The motor efferent fibers of the vagus nerve are located in the nucleus ambiguus; compression of the vagus nerve along its pathway can result in bilateral vocal cord dysfunction that typically presents as inspiratory stridor. Spontaneous recovery among patients with VCP has been observed most frequently among patients with underlying neurological or idiopathic pathology [11].

In a retrospective case review that identified a cohort of patients presenting with VCP, a Chiari malformation was the most common underlying neurological condition for VCP. Following surgical intervention, patients with VCP secondary to neurological disease had the highest rate of recovery within the VCP cohort [12].

Chiari type I malformation demonstrates ectopia or herniation of the cerebellar tonsils through the foramen magnum with symptomatic patients displaying at least 5 mm of herniation below the foramen magnum. Older age at diagnosis has been demonstrated to predict more severe symptoms. The surgical treatment of Chiari I has shown favorable outcomes in children [13]. Treatment for symptomatic Chiari I malformation is posterior fossa decompression with ventricular shunting in the presence of hydrocephalus. In a previous case study of a three-year-old presenting with stridor, bilateral vocal cord palsy, and a Chiari I malformation, posterior fossa decompression resulted in no resolution of vocal cord mobility, but stridor was absent at rest and during exertion during a three-month follow-up evaluation [14].

In a retrospective chart review of patients with VCP and Chiari malformation, four patients were identified as having VCP and Chiari I malformation. Of these, three achieved VCP resolution with two achieving resolution following neurosurgical decompression. Early decompression was associated with avoidance of tracheostomy. Early decompression may assist in reversing vocal cord dysfunction resulting from traction on the vagus nerve due to the downward displacement of the hindbrain [15].

Our case suggests that children with neurological diagnoses, including hydrocephalus and Chiari malformations, may have fluctuating stridor that is related to PVCM prior to developing bilateral vocal cord paresis and potentially requiring tracheotomy. Although reflux therapy is a known treatment for infantile PVCM, if an improvement is not seen over the course of months, this may suggest that vocal cord function may continue to decline in this population.

## Conclusions

This case of an infant with PVCM in the setting of Chiari II malformation with a VP shunt further supports that neurologic conditions can precipitate PVCM in infants. Further, the PVCM, in this case, recurred despite appropriate treatment of gastric reflux before progressing to bilateral vocal cord paralysis. In infants presenting with refractory PVCM, there should be a heightened awareness of the potential for progression to bilateral vocal cord paralysis and tracheostomy requirements.
